# Dickeya Manipulates Multiple Quorum Sensing Systems to Control Virulence and Collective Behaviors

**DOI:** 10.3389/fpls.2022.838125

**Published:** 2022-02-08

**Authors:** Fan Liu, Ming Hu, Zhijia Zhang, Yang Xue, Shanshan Chen, Anqun Hu, Lian-hui Zhang, Jianuan Zhou

**Affiliations:** Guangdong Laboratory for Lingnan Modern Agriculture, Guangdong Province Key Laboratory of Microbial Signals and Disease Control, Integrative Microbiology Research Center, South China Agricultural University, Guangzhou, China

**Keywords:** soft rot Pectobacteriaceae, *Dickeya*, quorum sensing, regulation, virulence

## Abstract

Soft rot Pectobacteriaceae (SRP), typical of *Pectobacterium* and *Dickeya*, are a class of Gram-negative bacterial pathogens that cause devastating diseases on a wide range of crops and ornamental plants worldwide. Quorum sensing (QS) is a cell-cell communication mechanism regulating the expression of specific genes by releasing QS signal molecules associated with cell density, in most cases, involving in the vital process of virulence and infection. In recent years, several types of QS systems have been uncovered in *Dickeya* pathogens to control diverse biological behaviors, especially bacterial pathogenicity and transkingdom interactions. This review depicts an integral QS regulation network of *Dickeya*, elaborates in detail the regulation of specific QS system on different biological functions of the pathogens and hosts, aiming at providing a systematic overview of *Dickeya* pathogenicity and interactions with hosts, and, finally, expects the future prospective of effectively controlling the bacterial soft rot disease caused by *Dickeya* by quenching the key QS signal.

## Introduction

Soft rot Pectobacteriaceae (SRP) belonging to the genera *Pectobacterium* and *Dickeya* (Charkowski et al., 2012) are emerging parasitic pathogens listed in the top ten important bacterial phytopathogens in the world (Mansfield et al., 2012). In addition to causing bacterial soft rot, these pathogens also cause blackleg of potato, stalk rot of maize, and foot rot of rice, resulting in considerable economic damage to vegetable and ornamental plant production worldwide. Previously, *Dickeya* was grouped into the genus *Erwinia* containing all plant-pathogenic Enterobacteriaceae, but in 2005, it was reclassified as a new genus *Dickeya* (Samson et al., 2005). Currently, twelve species are included in the *Dickeya* genus, including *Dickeya dianthicola*, *Dickeya dadantii*, *Dickeya zeae*, *Dickeya chrysanthemi*, *Dickeya paradisiaca*, *Dickeya solani*, *Dickeya aquatica*, *Dickeya fangzhongdai*, *Dickeya poaceaephila*, *Dickeya lacustris*, *Dickeya undicola*, and *Dickeya oryzae* (Samson et al., 2005; van der Merwe et al., 2010; Brady et al., 2012; Parkinson et al., 2014; van der Wolf et al., 2014).

Diverse phenotypic differentiation and complicated pathogenic mechanisms have been revealed in different *D. zeae* and *D. solani* strains. Recent studies compared the characteristics of *D. solani* strains isolated from countries with different climate conditions and found higher activities of cell wall degrading enzymes (CWDEs) and virulence in Polish strains than in Finland and Israel strains (Golanowska et al., 2017). *D. oryzae* and *D. zeae* strains isolated from rice, banana, and ornamental clivia in China showed different types of phytotoxins produced, including the zeamine I and zeamine II specific in some of the *D. oryzae* strains, as well as in *D. solani* strains (Zhou et al., 2011, 2015; Cheng et al., 2013; Hellberg et al., 2015), and another toxin produced by *D. zeae* banana strain MS2 but not the MS3 strain (Hu et al., 2018). Some *D. zeae* strains from different sources infect plant hosts in different ranges (Hu et al., 2018, 2021). Moreover, different structures of virulence factor modulating (VFM)-quorum sensing (QS) signals were implicated between *D. dadantii* 3937 and *D. oryzae* EC1 and *D. zeae* banana strains (Lv et al., 2019). Also, the functions of acyl-homoserine lactone (AHL) signals on the virulence of hosts are different in *D. oryzae* EC1 and *D. zeae* MS2 (Feng et al., 2019). The diversity of strains and the complexity of pathogenic mechanisms increase the difficulty of disease prevention and control in fields.

The achievement of the successful infection of *Dickeya* on plants depends on a complex range of virulence factors, including plant cell wall degrading enzymes (PCWDEs) (Hugouvieux-Cotte-Pattat et al., 2014), lipopolysaccharides (LPS), extracellular polysaccharide (EPS) (Condemine et al., 1992), iron carriers (Expert, 1999), pigment indigoidine, type III secretion system (T3SS) (Yang et al., 2002, 2004; Yap et al., 2005), and cell motility and adhesion associated with plants (Hussain et al., 2008; Chen et al., 2016, 2020). Motility, which is regulated by the AHL-QS signal, is a secondary virulence determinant of *Dickeya*, whereas pectinases in PCWDEs, which are regulated by the VFM-QS signal, are the primary virulence determinants of *Dickeya*, participating and dominating the macerating soft rot process of the pathogen in plant tissues. The capacity of synthesizing and secreting pectinases is modulated by complex and interconnected circuits involving multiple regulatory pathways. A recent study in our laboratory indicated that putrescine is a transkingdom communication signal modulating cell motility, biofilm formation, and virulence of *D. oryzae* EC1 (Shi et al., 2019). At the moment of current research, QS signals including the AHL signal, the VFM signal, and the crucial signal putrescine have been shown to participate in the intraspecific and transkingdom cell-cell communication, regulating the infection and the colonization of *Dickeya* toward host plants.

## *N*-Acyl-Homoserine Lactones-Quorum Sensing Signal Regulates Cell Motility and Biofilm Formation in *Dickeya*

Various gram-negative plant-pathogenic bacteria have been found to use a QS system dependent on the synthesis and perception of *N*-acyl-homoserine lactones (AHLs) as diffusible signals for coordinating QS communication. The AHL signal-mediated QS system is currently the most representative and most studied population sensing system, which is composed of two conserved categories of proteins in nearly all the sequenced *Dickeya* (except *D. paradisiaca*) (Potrykus et al., 2014) and *Pectobacterium* strains, ExpI (LuxI homolog) and ExpR (LuxR homolog). ExpI is a synthase in diverse *Pectobacterium* and *Dickeya* plant pathogens in charge of the synthesis of AHLs, including *N*-3-oxohexanoyl-homoserine lactone (3OC6-HSL), *N*-3-oxo-octanoyl-homoserine lactone (3OC8-HSL), *N*-hexanoyl-homoserine lactone (C6-HSL), and *N*-decanoyl-homoserine lactone (C10-HSL) in *Dickeya* (Nasser et al., 1998; Crépin et al., 2012; Feng et al., 2019), and *N*-octanoyl-homoserine lactone (C8-HSL), and *N*-3-oxo-decanoyl-homoserine lactone (3OC10-HSL) additionally in *Pectobacterium* (Crépin et al., 2012). ExpR is the AHL signal receptor and a transcriptional regulator modulating the expression of target genes.

In *Pectobacterium* and *Dickeya* bacteria, ExpR functions as a repressor in most cases, where ExpR binds to the promoter of DNA and blocks transcription in the absence of AHL, and AHL dissociates ExpR-DNA complexes to release ExpR and initiate transcription (Castang et al., 2006; Feng et al., 2019). For example, in *D. oryzae* EC1 and *D. zeae* MS2, the deletion of *expI* but not *expR* dramatically enhanced bacterial motility, aggregation, and pigment production (Hussain et al., 2008; Zhong, 2014; Feng et al., 2019). ExpR also represses its own transcription with a strong affinity for the *expR* regulatory region (Reverchon et al., 1998; Castang et al., 2006). Such autorepression will be relieved with the increase of AHL signal concentration. Unexpectedly, the inactivation of *expI* but not *expR* only affects the expression of *pelA* and *pelB* but does not result in visible repercussions on total pectate lyase activity in *D. dadantii* 3937 (Nasser et al., 1998), which also affects the expression of *prtX*, *prtB*, *prtC*, *pelB*, *pelE*, *pelL*, and some *zms* genes, and slightly reduces the production of pectinases and cellulases, and maceration on potato slices in *D. oryzae* EC1 (Hussain et al., 2008; Zhong, 2014). However, ExpR has been demonstrated to function as an activator to specifically interact with the promoters of the five major pectinase encoding genes *pelA*, *pelB*, *pelC*, *pelD*, and *pelE* (Nasser et al., 1998), and the *expI* in *D. dadantii* 3937 (Reverchon et al., 1998), and the addition of AHL signal dissociates ExpR-DNA complexes (Castang et al., 2006). In *D. dadantii* 3937, *D. oryzae* EC1, and *D. zeae* MS2, the deletion of *expR* has no effect on the virulence and the production of major virulence factors (Castang et al., 2006; Zhong, 2014; Feng et al., 2019). An exception is in *D. solani* that the *expR* mutant reduced the virulence on potato tubers by 2- to 3-fold compared with the wild-type strain (Potrykus et al., 2014), suggesting a certain difference in the degree of regulation on virulence genes by this system in *Dickeya* depending on the strain and host plant (Potrykus et al., 2018).

**FIGURE 1:**
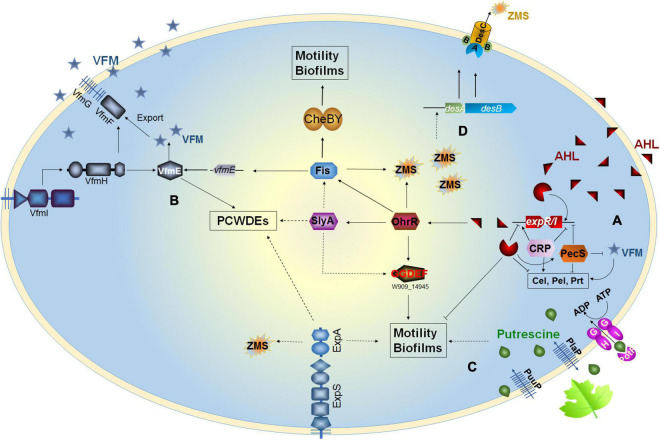
Regulatory pathways of the quorum sensing (QS) systems in *Dickeya*. **(A)** *Dickeya* bacteria produce AHL QS signal to regulate cell motility and biofilm formation; **(B)** VFM QS signal modulates the production of PCWDEs in *Dickeya*; **(C)** Putrescine is a transkingdom communication signal modulating cell motillity and biofilm formation; **(D)** Zeamines regulate the DesAB efflux pump.

In general, the ExpI–ExpR QS system plays an important role in bacterial motility, biofilm formation, and pigment production but has a limited role in the regulation of virulence traits. This system is also under the control of multiple cross-acting regulatory elements, such as cAMP-CRP and PecS in opposite manners (Figure 1A).

However, the ExpI–ExpR QS system has been shown to be quite critical in *Pectobacterium* species, which is involved in the production of PCWDEs and other virulence factors in *Pectobacterium* (Barnard and Salmond, 2007; Liu et al., 2008). There are several virulence regulators in *Pectobacterium* that act through the Rsm system, which plays a key role in controlling virulence, and the ExpI–ExpR QS system control occupies a vital position in the *Pectobacterium* regulatory hierarchy, with multiple downstream regulators, such as some that may operate through the Rsm system, also under the ExpI–ExpR QS system control.

Some studies reveal that AHLs produced by bacteria also act as interkingdom signals which influence and reprogram plant gene expression and also interact with other microbes (Mathesius et al., 2003; Bauer and Mathesius, 2004; Schuhegger et al., 2006; OrtÍz-Castro et al., 2008; von Rad et al., 2008; Schikora et al., 2011; Schenk et al., 2012).

Apart from the *luxR* genes paired with the adjacent *luxI* genes, many bacteria harbor *luxR* genes without *luxI* genes in their vicinity on the chromosome, called orphan or solo *luxR* (Hudaiberdiev et al., 2015). These solo *luxR* may respond to either internal AHL signals such as the *qscR* of *Pseudomonas aeruginosa* (Lequette et al., 2006) or exogenous signals such as the *sdiA* of *Escherichia coli* and *Salmonella enterica* (Ahmer, 2004; Hughes et al., 2010). In *D. zeae* MS2, the solo *luxR* (*pipR*) is linked to a proline iminopeptidase encoding gene *pipA* (Feng et al., 2019), similar to the solo *luxR* homologs in *Xanthomonas*, *Pseudomonas*, and *Kosakonia* (Zhang et al., 2007; Ferluga and Venturi, 2009; Coutinho et al., 2018; Mosquito et al., 2020). The expression of *pipA* is under the control of PipR, and the deletion of the *pipA*, but not *pipR*, in MS2 strain fully impaired its virulence on banana seedlings (Feng et al., 2019). In *Dickeya* genus, the *luxR*-solo systems, containing the *pipA/pipR* and the ABC-type peptide transporter genes (Feng et al., 2019), are only present in some strains including *D. parazeae* Ech586, 6 strains of *D. zeae*, 2 strains of *D. fangzhongdai*, *D. dadantii*, and *D. dianthicola*, respectively, 6 strains of *D. solani*, and *Dickeya* sp. Secpp 1600 (Table 1). Interestingly, this system is highly conserved in many *Klebsiella* strains but absent in the closely related species of *D. oryzae* (Table 1). Notably, all the host plants of *D. parazeae* and *D. zeae* harboring this *luxR*-solo system belong to monocotyledons (Table 1), suggesting that the system may be related to host specialization.

**TABLE 1:** Homologs of the *luxR*-solo systems (*C1O30_RS14500* to *C1O30_RS14535*) of *Dickeya zeae* MS2.

Strain	Country	Host	Coverage	Identity
*Dickeya zeae* MS2	Guangzhou	Banana	100%	100%
*Dickeya parazeae* Ech586	NA	*Philodendron*	100%	97.76%
*Dickeya zeae* A586-S18-A17	France: Durance River	River water	100%	97.38%
*Dickeya zeae* JZL7	China: Guangzhou	*Clivia minita*	100%	96.24%
*Dickeya zeae* PL65	United States: Hawaii	Taro	100%	96.04%
*Dickeya zeae* A5410	United States: Hawaii	Pineapple	100%	95.12%
*Dickeya zeae* CE1	China: Meizhou	*Canna edulis*	98%	96.48%
*Dickeya fangzhongdai* DSM 101947^T^	Zhejiang	*Pyrus pyrifolia*	90%	72.52%
*Dickeya fangzhongdai* ND14b	Malaysia	Waterfall	90%	72.47%
*Dickeya solani* IFB0223	Germany	Potato rhizosphere	90%	72.13%
*Dickeya solani* IFB 0099	Poland	Potato	90%	72.13%
*Dickeya solani* PPO 9019	Netherlands	Muscari	90%	72.13%
*Dickeya solani* D s0432-1	Finland	Potato stem	90%	72.13%
*Dickeya solani* RNS 08.23.3.1.A	France	Potato	90%	72.13%
*Dickeya solani* IPO 2222^T^	Netherlands	Potato	90%	72.13%
*Dickeya dadantii* DSM 18020^T^	Comoros	*Pelargonium capitatum*	90%	71.79%
*Dickeya dadantii* 3937	NA	*Saintpaulia ionantha*	90%	71.52%
*Dickeya dianthicola* ME23	United States:Maine	Potato	86%	73.08%
*Dickeya dianthicola* RNS04.9	NA	Potato	86%	73.08%
*Dickeya* sp. Secpp 1600	Hunan	Radish	90%	72.51%
*Klebsiella oxytoca* 4928STDY7071292, 4928STDY7071186,	United Kingdom	Human fecal	94%	79.63%
4928STDY7387739,				−79.96%
4928STDY7387706,				
4928STDY7387738				
*Klebsiella michiganensis* BD177	NA	NA	94%	79.67%
*Klebsiella michiganensis* K518, K516	Zhejiang	Human fecal	94%	79.67%
*Klebsiella oxytoca* CAV1374	United States:Virginia	Human perirectal	94%	79.67%
*Klebsiella michiganensis* E718	Taiwan	Human	94%	79.67%
*Klebsiella michiganensis* F107	Fujian	Human sputum	94%	79.65%
*Klebsiella oxytoca* KONIH4, KONIH2	United States	Waste water	94%	79.64% 79.63%
*Klebsiella oxytoca* KONIH1	United States	Perirectal swab	94%	79.64%
*Klebsiella michiganensis* C52	Sydney	Human clinical sample	94%	79.61%
*Klebsiella michiganensis* AR375	NA	NA	94%	79.62%
*Klebsiella oxytoca* AR_0028	NA	NA	94%	79.60%
*Klebsiella michiganensis* M1	South Korea	Soil	94%	79.61%
*Klebsiella michiganensis* HKOPL1	NA	Panda fecal	94%	79.61%
*Klebsiella michiganensis* KCTC 1686	South Korea	NA	94%	79.61%
*Raoultella electrica* DSM 102253	Japan	Anodic biofilm of microbial fuel cell	94%	79.03%

## The *Dickeya*-Specific Virulence Factor Modulating Signal Regulates Plant Cell Wall Degrading Enzymes Production and Virulence

**FIGURE 2:**
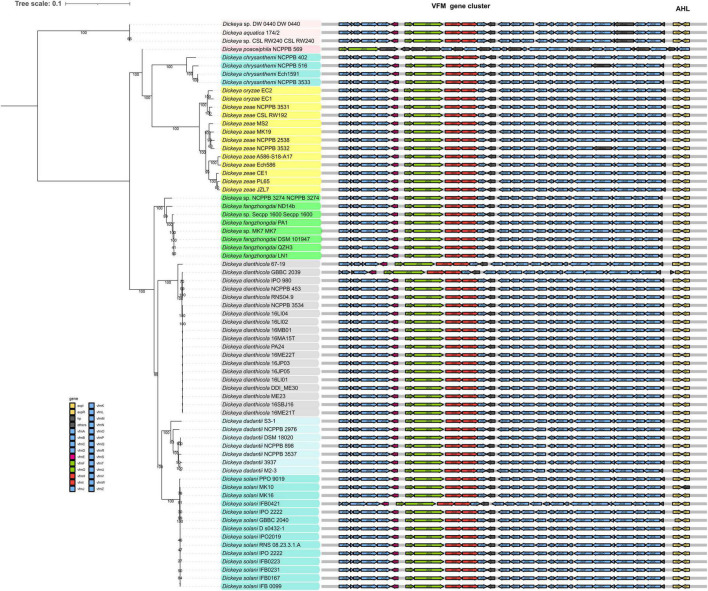
Phylogenetic analysis of 69 *Dickeya* spp. strains in NCBI RefSeq database based on 120 bacterial conserved single-copy genes. The virulence factor modulating (VFM)- and acyl-homoserine lactone (AHL)-QS gene clusters are located adjacent in the genomes. The genes labeled “others” are also located in the *vfm* gene cluster but share less similarity to the corresponding genes in EC1.

In 2013, Nasser et al. (2013) first identified the existence of a non-AHL-QS system in *D. dadantii* 3937, called the VFM system. This system is encoded by a 25-kb gene cluster, containing genes involved in the biosynthesis, transport, and induction of the VFM signal. With the exception of *D. poaceaephila* NCPPB 569 isolated from sugarcane, which had only 46% coverage, the *vfm* gene cluster is extremely conserved in all *Dickeya* strains whose whole genome sequences have been sequenced (Figure 2). Notably, this gene cluster is uniquely present in *Dickeya* strains, suggesting that the VFM-QS system is an inherent, ubiquitous, and unique regulatory mechanism in *Dickeya*. In all the tested strains such as *D. dadantii* 3937, four *D. solani* strains, *D. oryzae* EC1, and *D. zeae* MS2, the VFM-QS system has been demonstrated to participate in the regulation of PCWDE production and virulence (Nasser et al., 2013; Potrykus et al., 2014, 2018; Lv et al., 2019). Furthermore, in *D. oryzae* EC1, the VFM signal also regulates pathogen swimming and swarming motility and the production of phytotoxin zeamines (Lv et al., 2019).

Although the chemical structure of the VFM signal has not been characterized yet, some characteristics of this signal can be inferred through some experimental evidence. First, the signal is an extracellular compound since it can be sensed by the VfmI histidine kinase sensor using bacterial supernatant and activates the expression of the *vfmE*, encoding an AraC-type family transcriptional regulator through the VfmHI two-component system (Lv et al., 2019; Figure 1B). Second, the nature of the VFM signals produced by different *Dickeya* strains might be different. A *lacZ*-VFM reporter, constructed based on *D. oryzae* EC1 *lacZ* deletion mutant, could sense the VFM signals produced by EC1, *D. zeae* MS2 and MS3, but not strain *D. dadantii* 3937, *vice versa* (Lv et al., 2019), suggesting that the chemical structures of the VFM signals produced by EC1 and 3937 might be different. Moreover, the VFM signal from 3937 is stable after treatment by boiling at 100°C for 3 min (Nasser et al., 2013), while that from EC1 completely loses its activity after treatment at 50°C for 30 min (Lv, 2018). Third, the VFM signal is produced at the early and middle stages of bacterial exponential growth. During the growth of *D. oryzae* EC1, the production of VFM signal was quantified, and the results showed that when the cell density is lower than OD_600_ at 0.5, the VFM signal increased slowly with the increase of bacterial concentration; when the cell density is over OD_600_ at 0.5, the signal generation rate is obviously accelerated; after OD_600_ at 1.2, interestingly, the signal concentration decreased sharply (Lv, 2018), suggesting that VFM signal degrading enzymes are probably present in the genome of EC1. In addition, a *pecS* gene encoding a MarR family transcriptional regulator, along with the indigoidine biosynthesis gene *indABC*, is located upstream of the *vfm* gene cluster. These genes are mainly present in *Dickeya* spp. and absent in *Pectobacterium* spp. PecS functions as a repressor of the VFM system, and the deletion of *pecS* induces symptoms more rapidly than the wild-type strain (Hommais et al., 2008; Pédron et al., 2018), which may be attributed to the overproduction of VFM signal in the *pecS* mutant. PecS acts mainly in the early stages of infection, suggesting that it may prevent the *vfm* gene expression in the first stages of infection. Furthermore, VFM- and AHL-QS systems do not work in synergy to modulate the virulence of *Dickeya*. Previous studies have revealed that the AHL-QS system regulates the cell motility and biofilm formation of *Dickeya* spp., playing an essential role in pathogen colonization and survival in poor environmental conditions (Hussain et al., 2008; Feng et al., 2019), while the VFM-QS system is more dominant in regulating the production of diverse virulence factors and the ability to macerate plant tissue (Nasser et al., 2013; Lv et al., 2019). The interplay between these two QS systems has been studied in five *D. solani* strains at different virulence levels. The results showed that the two QS systems do not coordinate the virulence of *Dickeya* (Potrykus et al., 2018).

Apart from the PecS, Fis transcriptional regulator has been found to bind directly to the promoter region of *vfmE*, directly regulating the production of VFM signal and the PCWDEs (Lv et al., 2018). Recently, an organic hydroperoxide reductase regulator (OhrR) has been identified to directly bind to the promoter regions of *fis* and *slyA* (Lv et al., 2021). The two transcriptional regulators Fis and SlyA control the production of PCWDEs in completely different pathways (Zhou et al., 2016; Lv et al., 2018).

## Putrescine Acts as a New Type of Quorum Sensing and Transkingdom Communication Signal

Polyamines, mainly constituted by putrescine, spermine, spermidine, and cadaverine, are a group of aliphatic small polycationic molecules that can bind to RNA, DNA, nucleotide triphosphates, and other acidic substances, involved in regulating a wide variety of physiological processes within living organisms (Igarashi and Kashiwagi, 2000; Shi et al., 2019). In recent years, considerable evidence has demonstrated that in addition to core physiological functions, including translation, transcription, and chromatin remodeling, polyamines are involved in various fundamental cellular processes regulated by bacterial cells as QS signals. For instance, norspermidine acts as an intercellular signaling molecule that activates the biofilm formation of *Vibrio cholera via* a norspermidine sensor NspS-dependent manner, and the lack of norspermidine biosynthetic pathway results in severely reduced biofilm formation (Griffiths et al., 1984; Karatan et al., 2005; Lee et al., 2009). Spermidine transporter-dependent signaling pathway regulates the expression of the T3SS genes of *P. aeruginosa* (Zhou et al., 2007). Putrescine has been demonstrated to be critical for biofilm formation and motility in common human pathogens. In *E. coli*, putrescine interferes with biofilm formation and surface motility (Koski and Vaara, 1991; Kurihara et al., 2011). In *Yersinia pestis*, the deficiency of putrescine synthesis enzymes can severely inhibit biofilm formation, and the exogenous addition of putrescine can effectively rescue biofilm formation (Patel et al., 2006; Wortham et al., 2010). In the urinary tract pathogen *Proteus mirabilis*, putrescine serves as an extracellular signal essential for swarming motility and invasion ability (Sturgill and Rather, 2004; Kurihara et al., 2013).

In *Dickeya* spp., only putrescine and spermidine are produced since the spermine synthase encoding gene *spe4* is absent in the genomes. The previous study has shown that the deletion of the arginine decarboxylase encoding gene *speA* in *D. oryzae* EC1 impaired the synthesis of putrescine, bacterial motility, biofilm formation, and rice seed invasion ability (Shi et al., 2019). Putrescine can also act as an interkingdom communication signal transmitted through the bacterial plasma membrane by PotF and PlaP putrescine-specific transporters into the wild-type EC1 and *speA* mutant cells to activate and rescue the phenotype of bacterial motility (Shi et al., 2019; Figure 1C). In *D. zeae* MS3, the deletion of *speA*, but not *speE* (encoding spermidine synthase), decreases bacterial motility, biofilm formation, and virulence on banana seedlings and potato slices but has no effect on the production of PCWDEs (Tang, 2017), suggesting that putrescine, but not spermidine, is a conserved critical signal regulating the cell motility, biofilm formation, and virulence in *Dickeya*.

## Zeamines Modulate Toxin Production and Toxin Resistance in a Manner of Quorum Sensing Mechanism

Zeamines are polyamine phytotoxins produced by *D. oryzae* EC1, many *D. solani*, and some *Serratia* strains (Zhou et al., 2015). Zeamines are encoded by a *zms* gene cluster that includes 18 genes from *zms*O to *zms*N (Zhou et al., 2015). Among them, *zms*A is a key gene responsible for the biosynthesis of zeamines (Zhou et al., 2011), and *zmsK* encodes a non-ribosomal peptide synthase (NRPS) catalyzing the amide bond formation by using zeamine II as a substrate to generate zeamine (Cheng et al., 2013). The mutation of *zms*A and *zmsK* abolishes antimicrobial activity and attenuates the virulence of *D. oryzae* EC1 (Zhou et al., 2011; Cheng et al., 2013). Zeamines not only function as virulence factors in *D. oryzae* rice strains (Zhou et al., 2011; Cheng et al., 2013) but also antagonize many bacteria and fungi and even kill the nematode (Hellberg et al., 2015; Liao et al., 2015; Hu et al., 2018).

In EC1, the production of zeamines is under the control of multiple regulation pathways, such as the OhrR–SlyA–Fis, the OhrR–SlyA–Fis–VfmE, the VfmI–VfmH–VfmE, and the ExpS–ExpA (Zhou et al., 2016; Lv et al., 2018, 2019, 2021). Notably, an efflux pump DesABC specifically recognizes zeamines and is currently only found in many *Dickeya* spp. (Liang et al., 2019), implicating a mechanism of toxin tolerance in *Dickeya* bacteria. Surprisingly, zeamines also act as a regulator modulating the expression of *des*AB genes in a density-dependent manner. The exogenous addition of zeamines in a low concentration (5 μg/ml) could significantly induce the expression of *des*AB genes in *D. oryzae* EC1 and *D. dadantii* 3937 (Liang et al., 2019; Figure 1D), unveiling a novel and specific signaling role of zeamines in regulating microbial resistance to zeamines.

## Conclusion

Quorum sensing is a “language” for microbial communication to regulate the group behavior of microbes. The vital role of QS in bacterial virulence has attracted considerable interest from researchers, making it a promising novel target for the prevention and control of QS-mediated bacterial infections (Joshi et al., 2016). Such novel disease control strategy, called quorum quenching (QQ), is distinguished from other disease biocontrol measures in that QQ disrupts signal-mediated QS by inactivating QS signal or interfering with signal production or perception, not acting on the main growth factors of the pathogens; thus, it would not cause selective pressure on the survival of pathogens. In this study, QS systems that regulate the pathogenesis of *Dickeya* are systematically revealed which not only modulate the virulence of *Dickeya* but also affect the drug resistance and adaptability to the environment. They are differential in chemical structures, biosynthesis pathways, signal transduction pathways, and regulation mechanisms. For the widely conserved classical AHL-QS system, it mainly affects bacterial motility and biofilm formation and regulates the adaptability of *Dickeya* spp. to the surrounding environment. Except in *D. solani* strains that cause blackleg of potato disease in Western Europe, the AHL-QS system regulates the virulence of the pathogens (Potrykus et al., 2018). Given that VFM and putrescine are ubiquitous in *Dickeya* spp. and they function as major regulatory systems modulating virulence of *Dickeya* spp., we suggest focusing on quenching these two systems for prevention and control of bacterial soft rot on crops caused by *Dickeya.*

From current research results, no obvious evidence reveals that there is a relationship between AHL and VFM systems. However, since the VFM signal actions at a relatively low cell density (OD600 below 1.2), while the AHL signal functions when the cell density is high, we think that some gene(s) may be responsible for the switching between these two QS systems. For the putrescine signal, some of the regulon overlays with those regulated by the AHL signal, but no current evidence indicates any interplay between them. The interactions between different QS systems in *Dickeya* need more in-depth investigations to draw a more accurate and clear conclusion.

## Author Contributions

FL, MH, ZZ, YX, SC, AH, and JZ wrote the manuscript. L-HZ and JZ revised the manuscript. All authors contributed to the article and approved the submitted version.

## Conflict of Interest

The authors declare that the research was conducted in the absence of any commercial or financial relationships that could be construed as a potential conflict of interest.

## Publisher’s Note

All claims expressed in this article are solely those of the authors and do not necessarily represent those of their affiliated organizations, or those of the publisher, the editors and the reviewers. Any product that may be evaluated in this article, or claim that may be made by its manufacturer, is not guaranteed or endorsed by the publisher.
